# Human Decellularized Adipose Tissue Hydrogels as a Delivery Platform to Enhance Human Endothelial Colony-Forming Cell Retention and Vascular Regeneration

**DOI:** 10.3390/jfb17080368

**Published:** 2026-07-30

**Authors:** Agnes E. Terek, John T. Walker, Gillian I. Bell, John A. Ronald, Lauren E. Flynn, David A. Hess

**Affiliations:** 1 Department of Physiology and Pharmacology, Schulich School of Medicine and Dentistry, The University of Western Ontario, London, ON N6A 5C1, Canada; aterek@uwo.ca (A.E.T.); gbell22@uwo.ca (G.I.B.); 2 Robarts Research Institute, The University of Western Ontario, London, ON N6A 5C1, Canada; jronald@robarts.ca; 3 Department of Anatomy and Cell Biology, Schulich School of Medicine and Dentistry, The University of Western Ontario, London, ON N6A 5C1, Canada; jwalk29@uwo.ca; 4 Department of Medical Biophysics, Schulich School of Medicine and Dentistry, The University of Western Ontario, London, ON N6A 5C1, Canada; 5 School of Biomedical Engineering, Faculty of Engineering, The University of Western Ontario, London, ON N6A 3K7, Canada

**Keywords:** vascular regeneration, cell therapy, hydrogel, critical limb ischemia, decellularized adipose tissue (DAT), endothelial colony-forming cells (ECFC)

## Abstract

Cellular therapies harnessing the potential of endothelial colony-forming cells (ECFCs) for therapeutic revascularization in patients with critical limb ischemia (CLI) have garnered much interest. However, limited cell persistence following intramuscular injection into ischemic tissues has prompted the development of biomaterials as cell-delivery platforms to support cell survival and enhance therapeutic outcomes. This study explored the use of hydrogels derived from human decellularized adipose tissue (DAT) as a cell-instructive platform for ECFC delivery. In vitro studies confirmed high initial ECFC viability at 1 day following encapsulation in the DAT hydrogels but revealed that cell viability and proliferation were reduced over time in culture as compared to ECFCs cultured on tissue culture polystyrene (TCP). However, the ECFCs cultured within the DAT hydrogels showed similar ECFC cell surface marker expression patterns compared to ECFCs cultured on TCP after 6 days in culture, supporting that their phenotype was maintained. Subsequent testing focused on comparing a low (2.4 × 10^5^ cells) and high (1 × 10^6^ cells) dose of ECFCs delivered in either saline or within DAT hydrogels in a femoral artery ligation-induced CLI (FAL-CLI) model in NOD/SCID mice over 35 days. Bioluminescence imaging results showed that the low dose of ECFCs delivered in DAT hydrogels demonstrated enhanced retention on days 14, 21, and 28 compared to delivery in saline. Despite this finding, there were no observed improvements in hindlimb perfusion between delivery strategies, which may be related to the robust collateral vessel formation observed in this model with a µCT-based angiography method. Overall, this work supports that DAT hydrogels can enhance localized ECFC retention following intramuscular injection in mice with femoral artery ligation but emphasizes that enhanced retention may be insufficient for improving functional vascular regeneration.

## 1. Introduction

Peripheral arterial disease (PAD) is caused by atherosclerotic plaque formation and subsequent occlusion of arteries supplying blood to the lower limbs [1]. Globally, >235 million people have PAD, and its prevalence is rising dramatically given the increasing burden of risk factors including type 2 diabetes (T2D) and obesity in the aging population [2,3]. PAD can progress to occlusion with microvascular involvement, a condition also known as critical limb ischemia (CLI), characterized by ischemic pain at rest, non-healing ulcers, and gangrene [1]. Treatments for CLI include angioplasty and stent placement or bypass graft surgery. However, up to 20% of individuals with CLI are not eligible for surgical revascularization [4] and rely solely on symptom management. Due to the limited efficacy of current clinical approaches, approximately 25% of patients require amputation within one year of CLI diagnosis [5]. Amputation dramatically impairs quality of life and is associated with a 50% mortality rate at 1-year post-surgery [1].

The lack of effective therapies for CLI has motivated investigation of alternative treatment approaches, including cell-based therapies targeted at enhancing vascular regeneration [6,7]. With a central role in blood vessel formation, human endothelial colony-forming cells (ECFCs) are highly proliferative endothelial precursor cells that can differentiate into mature endothelial cells that form capillary-like tubules in vitro [8] and contribute to neovascularization by integration into blood vessels in vivo [9].

From a translational perspective, multiple clinical trials investigating delivery of autologous bone marrow-derived hematopoietic and endothelial cells have demonstrated safety and symptom relief; however, amputation rates remain unchanged [10]. Important factors believed to limit the clinical efficacy of cell therapies for CLI are the poor survival and low engraftment of transplanted cells [6]. Ischemic muscle tissue of CLI patients represents a harsh environment characterized by hypoxia, nutritional stress, alterations in the extracellular matrix (ECM), and pathological inflammation [11]. Recent bioengineering approaches designed to improve the survival of transplanted cells include the use of ECM-derived biomaterials to support cell engraftment and retention [12]. ECM-derived bioscaffolds generated from decellularized tissues can provide a three-dimensional (3D) microenvironment incorporating a complex array of collagens, glycoproteins and polysaccharides, as well as latent cytokines and pro-angiogenic growth factors [13]. Therefore, bio-scaffolds can provide structural support and bioactive cues to help maintain viability and direct the pro-angiogenic function of transplanted cell populations [14].

In particular, human adipose tissue has been identified as a readily available source of bioactive human ECM [15] that can be isolated through decellularization and processed to generate various decellularized adipose tissue (DAT) bioscaffold formats, including particulates, microcarriers, foams, and hydrogels, that can be used for both cell culture and delivery [16,17]. While primarily comprised of type I collagen, DAT is also a source of a range of growth factors, cytokines, and matricellular proteins [18]. Our previous work has shown that human DAT scaffolds seeded with adipose-derived stromal cells (ASCs) were well tolerated in vivo, integrated readily into host tissues, and supported endogenous angiogenic processes [19]. In addition, delivery on DAT foams was shown to enhance the survival and promote the pro-angiogenic function of human dermal fibroblasts compared to delivery on collagen foams in athymic nude mice [20]. Moreover, implantation of human hematopoietic progenitor cells in DAT scaffolds accelerated the recovery of limb perfusion and improved functional limb use in NOD/SCID mice following unilateral femoral artery ligation relative to DAT implantation alone or saline-injected controls [21]. Taken together, these studies suggest that through the collagens and array of signaling molecules contained within the decellularized matrix, DAT may provide both a structural and bioactive support for ECFCs. This is hypothesized to occur primarily through augmentation of the angiogenic potential of the donor ECFCs, as well as through the stimulation of endogenous stromal cells to support the development of stable vasculature.

Injectable hydrogels that enable minimally invasive delivery followed by in situ gelation represent a promising strategy for intramuscular cell delivery for the treatment of PAD/CLI [22]. Hydrogels have been widely explored for the delivery of ECFCs, using materials such as collagen type I, bovine fibrin, and synthetic peptide PuraMatrix^TM^ matrices, which have shown the ability to support rapid onset vascularization [23]. However, the delivery of ECFCs within DAT hydrogels has not yet been investigated. The primary goal of this study was to characterize DAT hydrogels as a delivery platform for human ECFCs, including in vitro studies to assess ECFC viability, proliferation, and phenotype following encapsulation in human DAT hydrogels. Subsequently, in vivo testing was performed to assess human ECFC delivery at two doses in DAT hydrogels on the retention of viable cells and restoration of perfusion in the ischemic hindlimb of NOD/SCID mice.

## 2. Materials and Methods

### 2.1. ECFC Isolation

Umbilical blood cord samples were collected at the London Health Sciences Birthing Centre with Research Ethics Board approval from Western University (HSREB#12934). ECFCs were established according to previously published protocols [24] and expanded in complete endothelial growth medium (EGM-2; Lonza, Walkersville, MD, USA). ECFCs were maintained in a humidified incubator at 37 °C and 5% CO_2_ and passaged every 3 days using TrypLE^TM^ Express (Gibco, Grand Island, NY, USA). ECFCs for all experiments were used between passage 5 and 9.

### 2.2. DAT Hydrogel Preparation and Cell Encapsulation

DAT hydrogels were synthesized from human subcutaneous fat harvested with informed consent from patients receiving elective surgical reduction of the abdomen or breast at the London Health Sciences Center (HSREB#105426). Tissue decellularization was performed using an established 5-day detergent-free protocol [15]. Two batches of DAT were used across all experiments, one from 5 pooled adipose tissue donors and one from 7. Following decellularization, DAT was milled and rinsed with ethanol before being suspended at a concentration of 25 mg/mL in 1 mg/mL porcine pepsin (Sigma-Aldrich, St. Louis, MO, USA) in 0.05 M hydrochloric acid and digested at room temperature for 24 h. DAT solution was then stored in the fridge at 4 °C until use.

For encapsulation, pepsin-digested DAT solution was neutralized and diluted to a concentration of 17.5 mg/mL using EGM-2 containing ECFCs at a density of 1.2 × 10^7^ cells/mL hydrogel solution. For in vitro studies, 100 μL of hydrogel formulation was loaded into a 0.3 mL syringe mold and placed at 37 °C for 2 h to permit gelation prior to ejection into complete EGM-2 media.

### 2.3. ECFC Survival, Proliferation and Phenotype In Vitro

ECFC distribution and viability following hydrogel encapsulation were assessed over 6 days in culture using the Live/Dead^®^ assay kit (ThermoFisher, Waltham, MA, USA) imaged on a Zeiss LSM confocal microscope. Gel contraction was quantified by hydrogel surface area analyses using ImageJ (version 1.53). ECFCs were extracted from hydrogels for flow cytometry analyses through enzymatic digestion with 1000 U/mL collagenase type IV (Fisher Scientific, Waltham, MA, USA) for 2 h at 37 °C under agitation, with TrypLE^TM^ Express (Gibco) added in for the last 10 min. Contents were filtered through 50 μm cell strainers (CellTrics, Görlitz, Germany) to remove large ECM fragments. Cells were detached from TCP via incubation with TrypLE only. ECFC lines were donor-matched for TCP and hydrogel comparisons. Cells were identified and singlets selected based on the forward and side scatter parameters. ECFC viability was assessed using Zombie Yellow^TM^ fixable viability kit (BioLegend), and ECFC proliferation was detected after a 24-h EdU pulse using the Click-iT^TM^ EdU Alexa Fluor^TM^ 488 Flow Cytometry Assay Kit (Fisher Scientific). Flow cytometry was also used to assess ECFC phenotype measuring cytosolic aldehyde dehydrogenase (ALDH) activity using ALDEFLUOR^TM^ kits (Stem Cell Technologies, Vancouver, BC, Canada), as well as CD31, VEGFR2, CD34, and CD133 expression at 1 and 6-days post-encapsulation in DAT, Geltrex^TM^, and OptiCol^TM^ hydrogels. Cell surface markers were detected using the following antibody dilutions: 1:40 CD34 AF700 (BioLegend, San Diego, CA, USA), 1:50 CD133 PE (Miltenyi Biotec, Bergisch Gladbach, Germany), 1:40 CD31 FITC (BD Biosciences, Franklin Lakes, NJ, USA), and 1:50 VEGFR2 PE-Dazzle 594 (BioLegend, San Diego, CA, USA). Gates were again drawn on cells followed by singlets before assessment of surface marker expression. Flow cytometry was performed on a BD LSRII at the London Regional Flow Cytometry Facility.

### 2.4. Lentiviral Transduction

To enable longitudinal cell tracking in the ischemic hindlimb model in vivo, lentiviral transduction methods were adapted to generate ECFCs expressing dTomato (dT) and the bioluminescent protein firefly luciferase (FLuc2) under the human elongation factor 1-α promoter [25]. Briefly, ECFCs were seeded in endothelial basal media (Lonza) with 8 μg/mL of polybrene and the lentivirus at a multiplicity of infection of 2 and incubated for 6 h at 37 °C before addition of complete EGM-2 media, followed by overnight incubation. Transduction efficiency (>80%) was confirmed via flow cytometry using the dT reporter. Flow cytometry was performed on transduced ECFCs to assess ECFC viability, proliferation, and CD31^+^ expression compared to non-transduced ECFCs.

### 2.5. Murine Femoral Artery Ligation-Induced Critical Limb Ischemia

All animal procedures were performed in accordance with regulations by the Canadian Council on Animal Care, as per animal use protocol #2023-031, approved by the Animal Care Committee at Western University. All in vivo experiments used 8–11-week-old NOD.CB17-Prkdcscid/J non-obese diabetic/severe combined immune deficiency (NOD/SCID) mice (Jackson Labs, Bar Harbor, ME, USA). Both male and female mice were used and assigned to treatment groups for a total of 6–9 mice per group.

Unilateral hindlimb ischemia by ligation and excision of a portion (~3 mm) of the femoral artery and vein in the right hindlimb was performed following established protocols [26,27,28]. Laser Doppler perfusion imaging (LDPI) was performed on day 1 post-surgery prior to transplantation to confirm that the perfusion ratio in the ischemic-versus-non-ischemic limb was <0.15. Each mouse was randomly assigned to one of 6 treatment groups: (1) 2.4 × 10^5^ ECFCs in DAT hydrogel, (2) 1 × 10^6^ ECFCs in DAT hydrogel, (3) 2.4 × 10^5^ ECFCs in saline, (4) 1 × 10^6^ ECFCs in saline, (5) DAT hydrogel alone, and (6) saline alone. Under anesthesia, 20 μL was injected intramuscularly into the right hindlimb just distal to the site of surgery.

### 2.6. Bioluminescence Imaging

Longitudinal bioluminescence imaging (BLI) assessments of ECFC retention in the ischemic limb were performed on post-surgical day 1 (immediately following cell injection), 3, 7, 14, 21, 28, and 35 using an IVIS XRMS In Vivo Imaging System (PerkinElmer, Shelton, CT, USA). Anaesthetized mice received an intraperitoneal injection of 100 µL of D-luciferin (30 mg/mL) in PBS (Syd Labs, Boston, MA, USA). Images were taken using autoexposure until the average radiance reached peak value. A region of interest (ROI) of consistent size was drawn around the site of injection and used to determine average radiance (p/s/cm^2^/sr) of the luminescent signal.

### 2.7. Laser Doppler Perfusion Imaging (LDPI)

Measurement of hindlimb perfusion was performed on post-surgical days 1, 3, 7, 14, 21, 28, and 35 using LDPI. Mice were anesthetized and warmed to maintain internal temperature between 36–38 °C using a CODA Monitor system (Kent Scientific, Torrington, CT, USA). Mice were imaged in a supine position using a Moor LDI2-HIR laser Doppler imaging system (Moor Instruments, Devon, UK). Average flux in the ischemic-versus-non-ischemic limb was quantified in a ROI consisting of the foot and ankle joint. Perfusion ratio was calculated as the average flux in the ischemic limb divided by the average flux in the control limb normalized to the perfusion ratio on day 1.

### 2.8. Immunofluorescence Staining of CD31^+^ Cell Density in the Ischemic Muscle

Immunofluorescence staining of gastrocnemius muscle sections was performed to quantify the CD31^+^ cell density within both the ischemic (surgical) and non-ischemic (control) limbs on day 35 post-ligation. Tissues were fixed in 10% buffered formalin, embedded in OCT compound and frozen at −80 °C. Tissue sections (10 μm) were co-stained with primary antibodies for CD31 (1:50 in blocking solution, goat polyclonal anti-human/mouse/rat CD31, Bio-Techne^®^, AF3628, Minneapolis, MN, USA) and α-smooth muscle actin (1:100 in blocking solution, rabbit polyclonal anti-human/mouse α-SMA, Abcam, ab5694, Waltham, MA, USA). The secondary antibodies were donkey anti-rabbit IgG Alexa FluorTM 647 (1:500 in blocking solution, Invitrogen, Carlsbad, CA, USA) and donkey anti-goat IgG Alexa Fluor^TM^ 488 (1:500 in blocking solution, Invitrogen). The Ready Probes™ Tissue Autofluorescence Quenching Kit (Invitrogen, Carlsbad, CA, USA) solution was applied according to the manufacturer’s protocol and slides were mounted using DAPI-enhanced mounting medium (Abcam, Waltham, MA, USA). Cross-sections of the muscle tissues were imaged on an Axio Imager Z2 (Zeiss, Oberkochen, Germany) at 10 X magnification. For each mouse, 3 sections were analyzed that were a minimum of 100 mm apart, and each section was imaged at 3 random, non-overlapping fields. Fiji imaging software was used to quantify the CD31 positive signal area over the total area of the section and normalized to the ratio for the control (non-surgical) limb.

### 2.9. Micro-Computed Tomography Angiography of Mouse Hindlimb Vasculature

To visualize vessel adaptations within the ischemic hindlimb, we performed micro-computed tomography (μCT) to detect perfused vasculature surrounding the surgical site [29]. Mice were perfused with Microfil^®^ Silicone Rubber (MV-122 Yellow) Injection Compound (Flowtech Inc., South Windsor, CT, USA) and fixed in 10% buffered formalin. μCT images were acquired using a GE eXplore Locus scanner. 3D Slicer image computing was used to generate 3D models of the hindlimb vasculature.

### 2.10. Statistical Analysis

All data was expressed as the mean ± standard deviation (SD). All comparisons were assessed for significance using one-way or two-way analysis of variance (ANOVA) with Tukey’s post hoc test, using GraphPad Prism version 10. Differences were considered statistically significant at *p* < 0.05.

## 3. Results

### 3.1. ECFC Viability, Proliferation, and Surface Marker Expression Following Culture in Hydrogels

**Figure 1:**
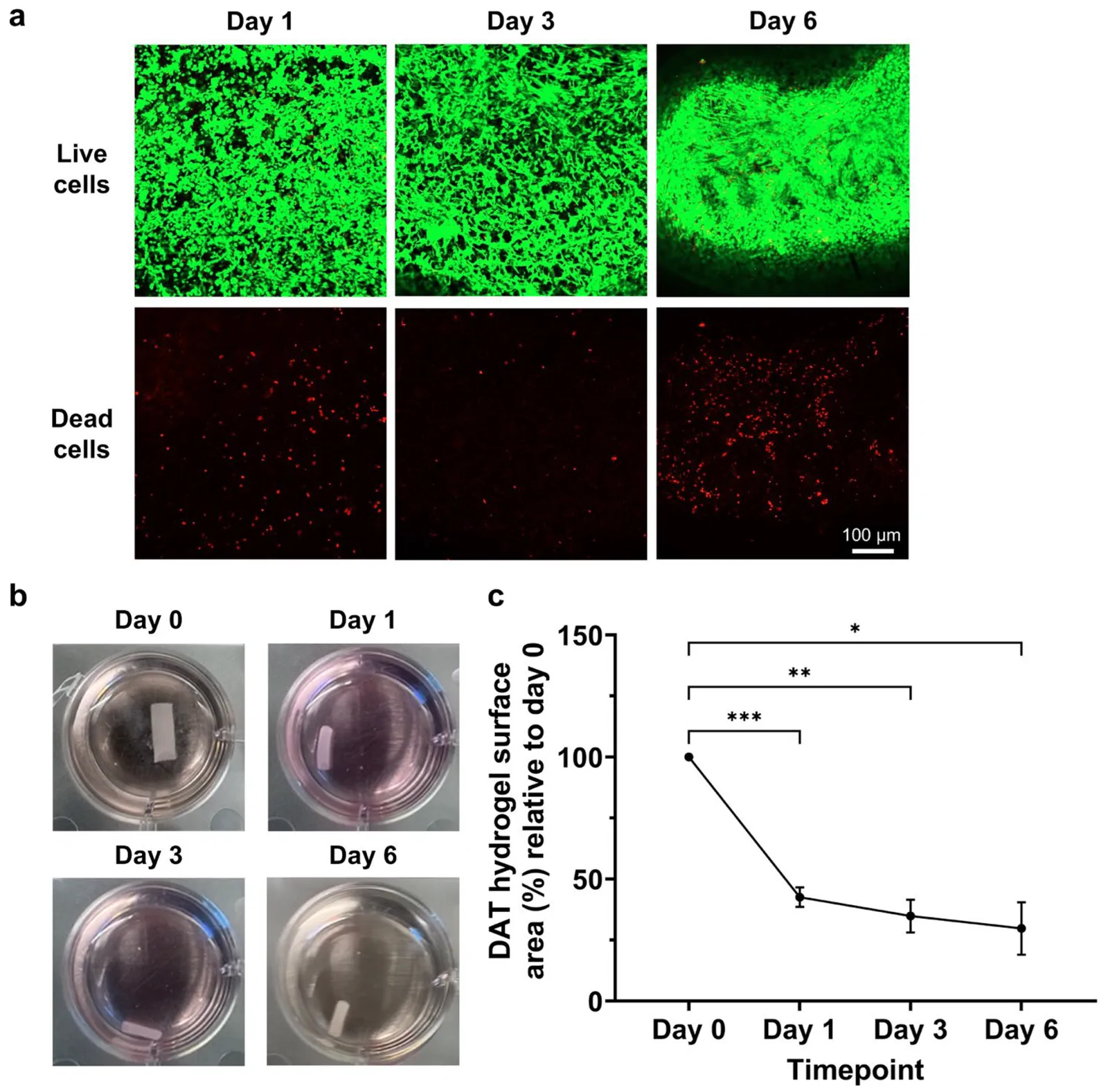
Human ECFCs encapsulated in DAT hydrogels remained viable in culture with gel contraction observed over time. (**a**) ECFCs encapsulated in DAT hydrogels with live cells shown in green (calcein AM) and dead cells shown in red (ethidium homodimer-1). (**b**) Representative macroscopic images of hydrogel contraction after ECFC encapsulation and culture for up to 6 days. (**c**) Quantification of ECFC-mediated hydrogel contraction (N = 3–4 cell lines per group). * *p* < 0.05, ** *p* < 0.01, *** *p* < 0.001, vs. day 0.

ECFCs were successfully encapsulated within the DAT hydrogels, with confocal analysis of LIVE/DEAD™ staining revealing high cell viability within the hydrogels, with only a few dead cells detected (Figure 1a). Notably, over the 6-day culture period, the DAT hydrogels containing encapsulated ECFCs displayed significant graft contraction (Figure 1b,c).

**Figure 2:**
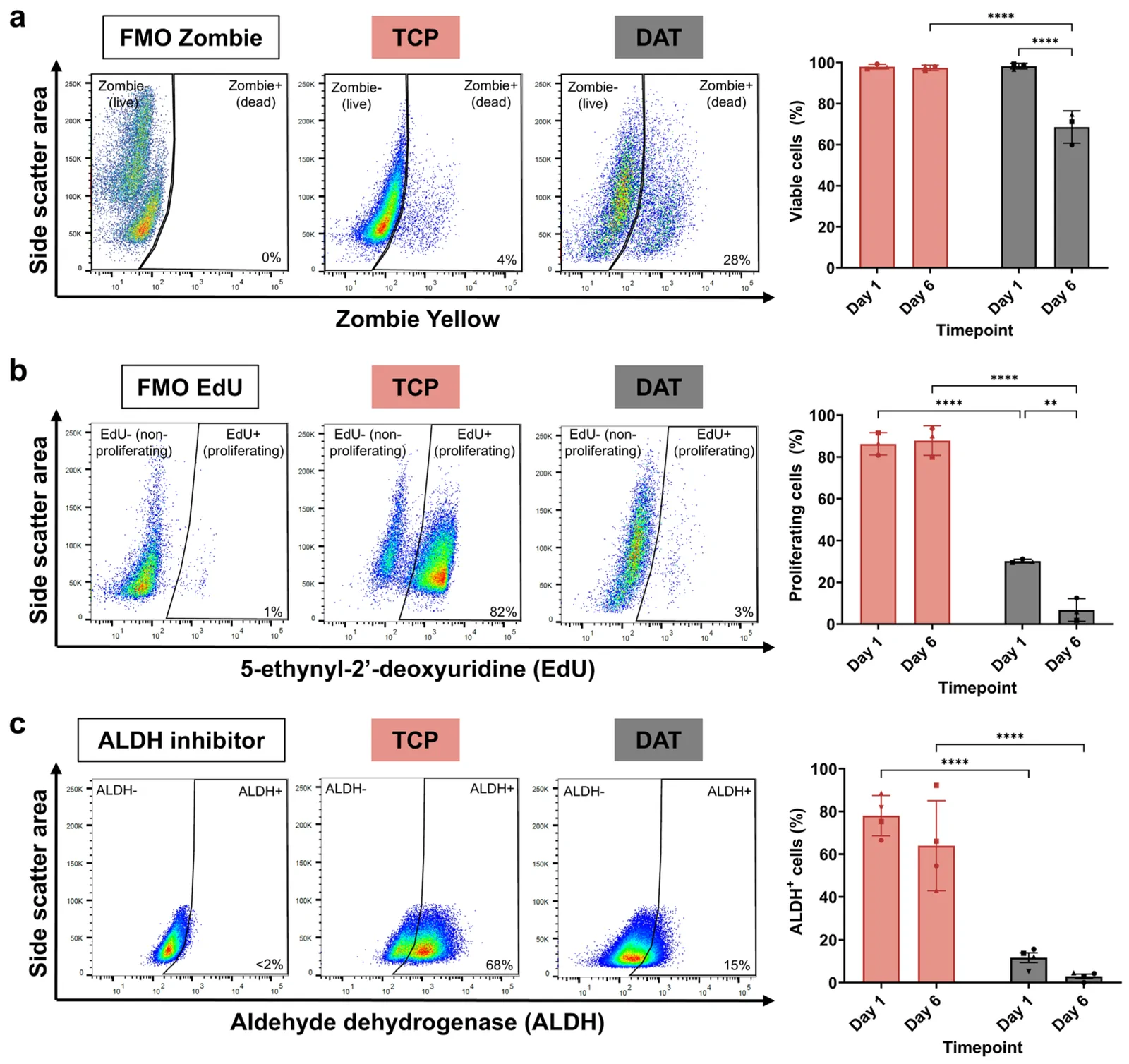
Encapsulation and culture of ECFCs within DAT hydrogels altered ECFC viability, proliferation, and ALDH-activity. (**a**) Representative flow cytometry plots showing Zombie Yellow FMO control incorporating heat-shocked cells, and ECFCs grown on TCP or DAT hydrogels for 6 days with quantification of viable ECFCs. (**b**) Representative flow cytometry plots showing EdU FMO control, and ECFCs grown on TCP or DAT hydrogels for 6 days with quantification of frequency of proliferating (EdU^+^) ECFCs. EdU-incorporation was detected using the Click-it^TM^ flow cytometry kit. (**c**) Representative flow cytometry plots showing ALDH-activity with inhibition using DEAB, and ALDH-activity for ECFCs grown on TCP or DAT hydrogels for 6 days with quantification of ECFCs with high ALDH activity. ALDH-activity was quantified using the Aldefluor^TM^ flow cytometry kits. (N = 3–4 cell lines per group). ** *p* < 0.01, **** *p* < 0.0001.

Flow cytometry was used to quantify ECFC viability (Zombie Yellow) and proliferation (EdU) after culture in the DAT hydrogels compared to on TCP (Figure 2a,b). ECFC viability was high (>80%) in the DAT hydrogels on day 1, but viability was significantly reduced by day 6 (Figure 2a). The frequency of proliferating ECFCs demonstrated a significant reduction between days 1 and 6 in the DAT hydrogels, and both days showed a reduced frequency of proliferating cells compared to culture on tissue culture plastic (TCP) (Figure 2b). ALDH-activity, a cytoprotective enzyme function conserved in hematopoietic and endothelial precursor cells [27], ranged between 60–80% on TCP, and was significantly reduced for ECFCs cultured in DAT hydrogels on both days 1 and 6 (Figure 2c).

**Figure 3:**
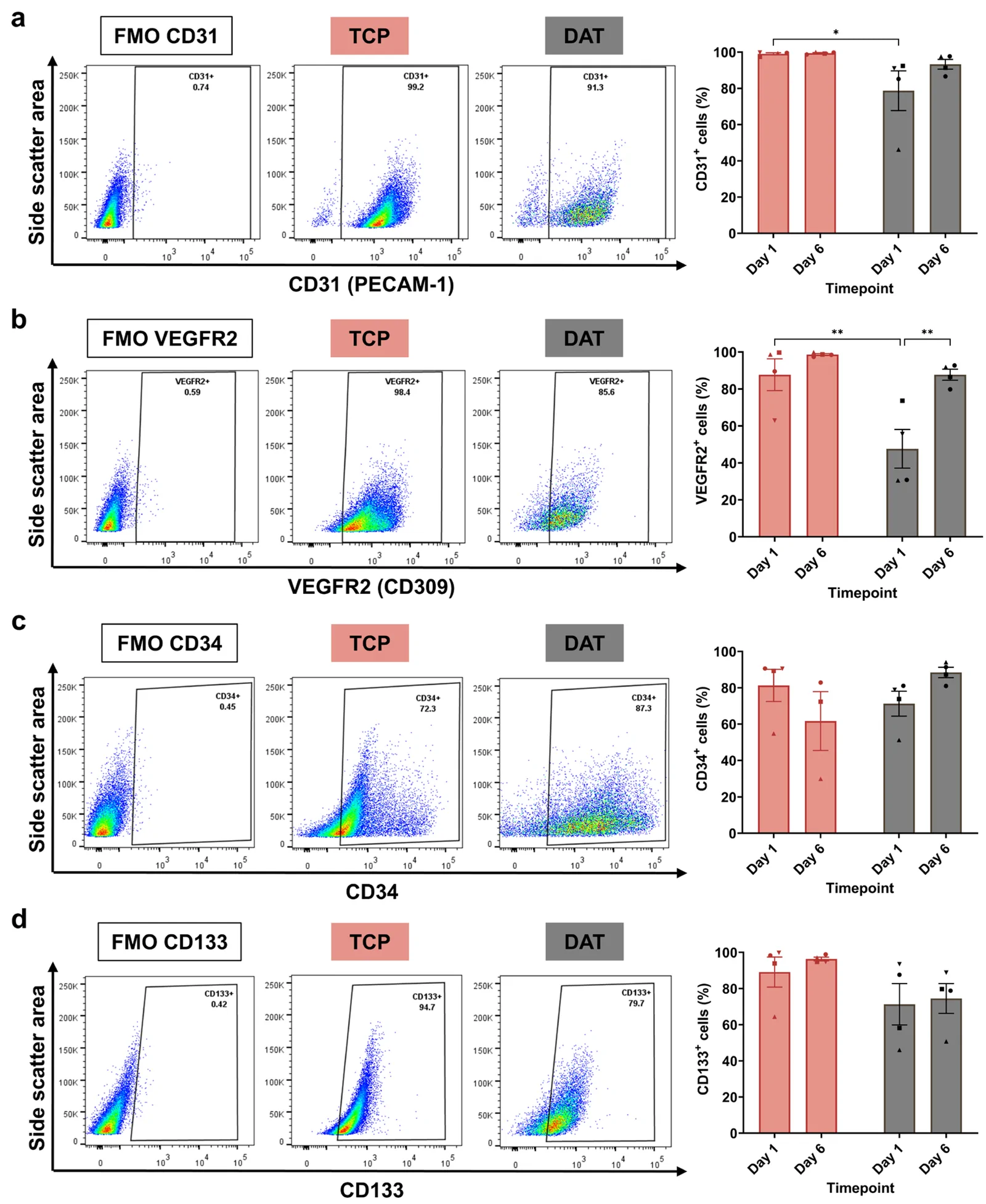
Encapsulation and culture of ECFCs within DAT hydrogels altered endothelial cell marker expression. (**a**) Representative flow cytometry plots showing the CD31 FMO control, and ECFCs grown on TCP or DAT hydrogels for 6 days with quantification of CD31^+^ ECFCs. (**b**) Representative flow cytometry plots showing the VEGFR2 FMO control, and ECFCs grown on TCP or DAT hydrogels for 6 days, with quantification of VEGFR2^+^ ECFCs. (**c**) Representative flow cytometry plots showing CD34 FMO control, and ECFCs grown on TCP or DAT hydrogels for 6 days, with quantification of CD34^+^ ECFCs. (**d**) Representative flow cytometry plots showing the CD133 FMO control, and ECFCs grown on TCP or DAT hydrogels for 6 days, with quantification of CD133^+^ ECFCs. (N = 3–4 cell lines per group). * *p* < 0.05, ** *p* < 0.01.

ECFCs cultured in the DAT hydrogels or on TCP were also analyzed for expression of the pan-endothelial cell marker CD31 and for expression of markers associated with endothelial precursors (VEGFR2, CD34, and CD133) (Figure 3). ECFC expression of CD31 remained >80% for both the DAT hydrogels and TCP on days 1 and 6 (Figure 3a). ECFC expression of VEGFR2 was significantly reduced in DAT hydrogels compared to TCP on day 1, but was restored to the levels seen on TCP at day 6 (Figure 3b). There were no significant differences in CD34 expression for ECFCs cultured in DAT hydrogels compared to TCP at both days 1 and 6 (Figure 3c). Similarly, there were no significant differences in CD133 expression for ECFCs cultured in DAT hydrogels compared to TCP at both timepoints (Figure 3d).

### 3.2. In Vivo Assessment of DAT Hydrogels as a Delivery Platform for ECFCs

**Figure 4:**
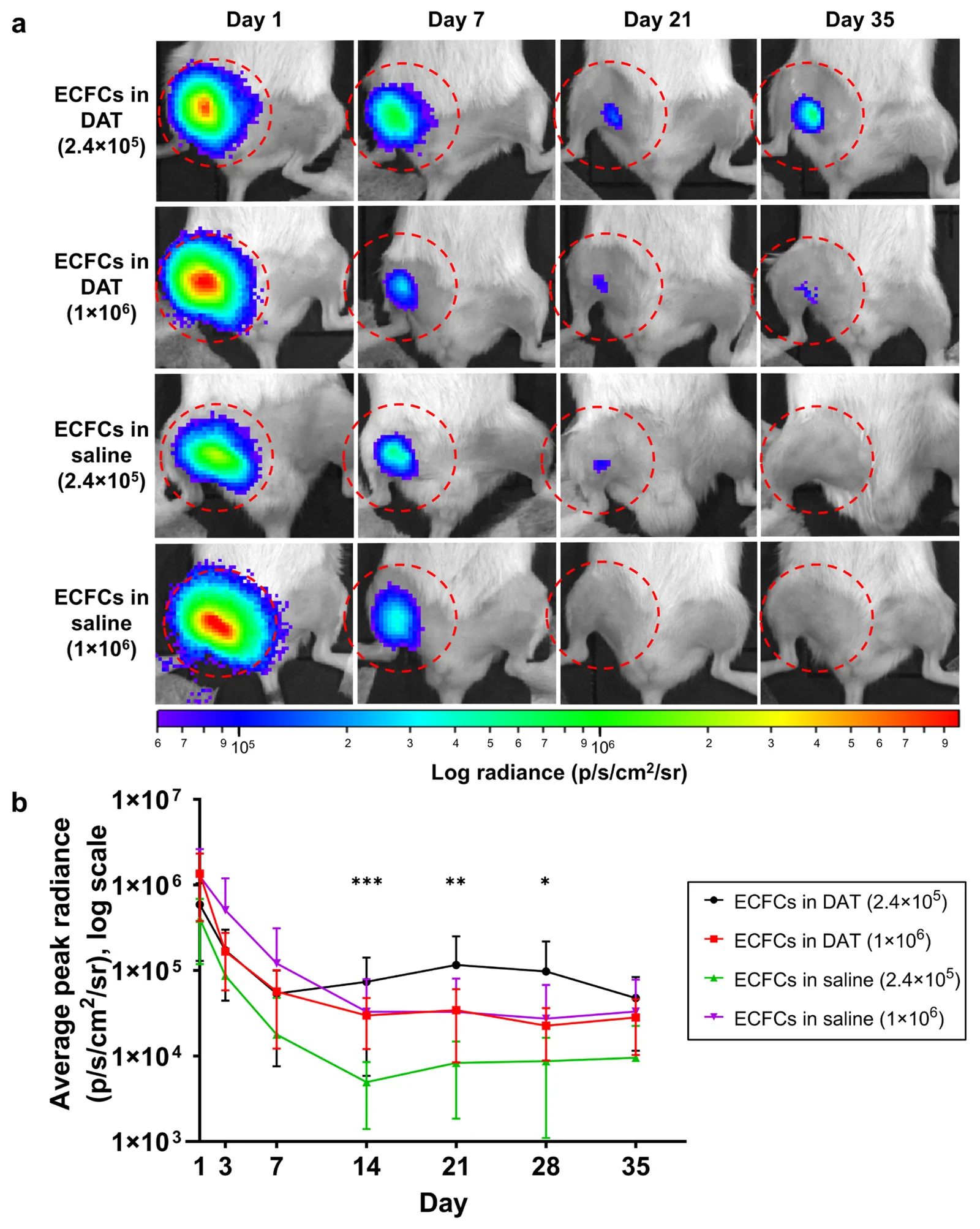
Delivery of a low dose of ECFCs in DAT hydrogels enhanced cellular retention compared to a low dose in saline. (**a**) Representative bioluminescence imaging on days 1, 7, 21, and 35 after ECFC injection in DAT hydrogels or in saline at either low or high dose. (**b**) Quantification of average peak radiance in the region of interest at the injection site (N = 6 cell lines; n = 6–7 mice/group). * *p* < 0.05, ** *p* < 0.01, *** *p* < 0.001 for ECFCs in DAT (2.4 × 10^5^) vs. ECFCs in saline (2.4 × 10^5^).

Immunodeficient NOD/SCID mice with femoral artery ligation-induced limb ischemia were injected with ECFC-seeded DAT hydrogels at multiple ECFC doses to assess cellular retention and recovery of perfusion in comparison to ECFC delivery in saline as the current standard practice. ECFCs expressing dT and the bioluminescent reporter FLuc2 were generated via lentiviral transduction (Figure S1), and transduced ECFCs were detected by BLI to assess cellular retention following IM-delivery in the ischemic limb. The bioluminescent signal on day 1 following cell injection was bright and well localized (Figure 4a) and rapidly declined over the first 7 days (Figure 4b). Enhanced cellular retention was demonstrated with the low dose of 2.4 × 10^5^ ECFCs delivered in DAT hydrogels on days 14 through 28 compared to low-dose ECFCs delivered in saline (Figure 4b; *p* < 0.05).

**Figure 5:**
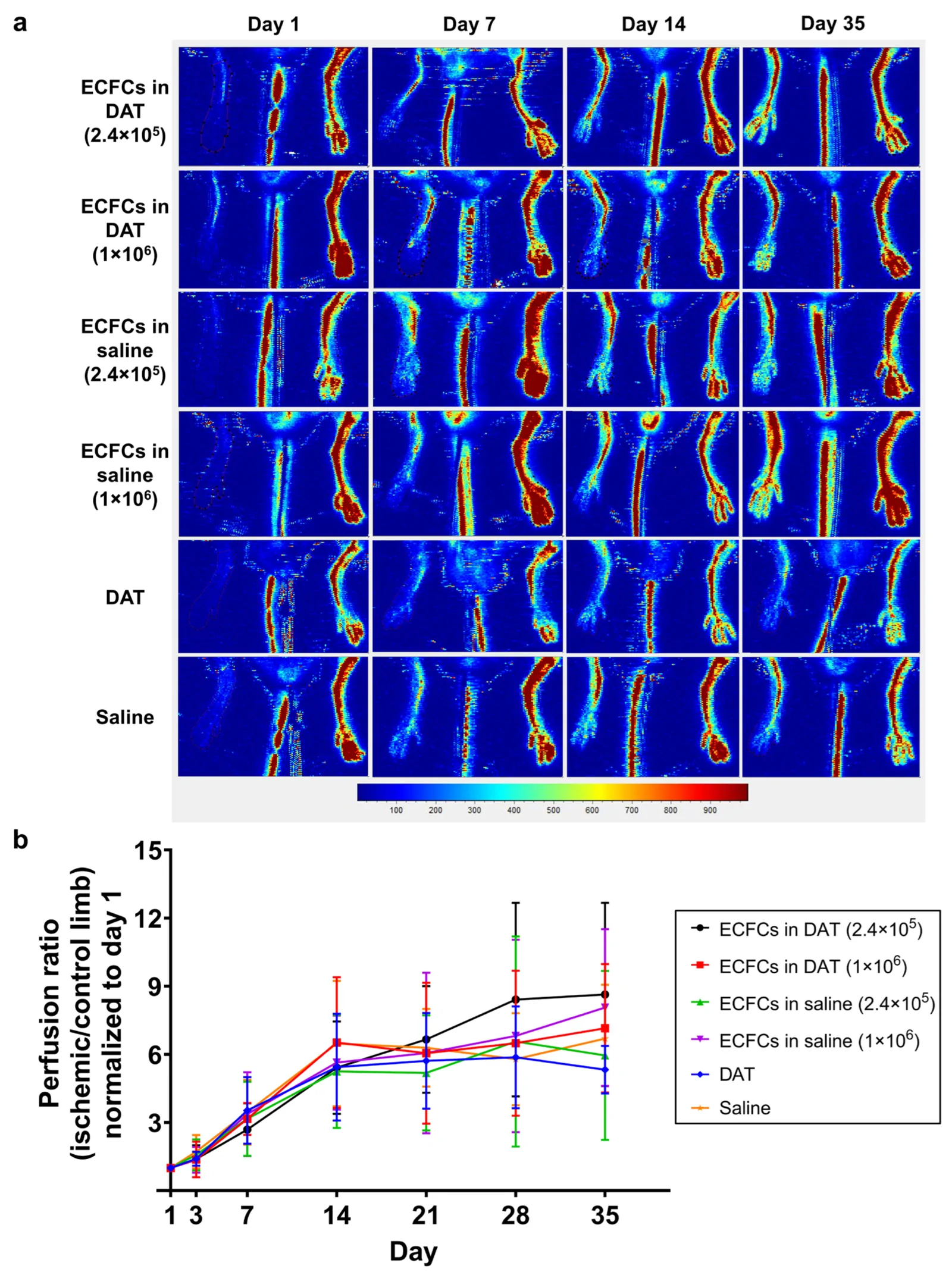
Recovery of limb perfusion following ECFC delivery in DAT hydrogels or saline. (**a**) Representative laser Doppler perfusion imaging on days 1, 7, 14, and 35 after femoral artery ligation and ECFC injection on day 1 at either low or high dose. (**b**) Quantification of mean flux in a region of interest comprising the foot and ankle, with a perfusion ratio calculated as ischemic/control limb normalized to day 1 (N = 6 ECFC lines; n = 6–9 mice/group).

**Figure 6:**
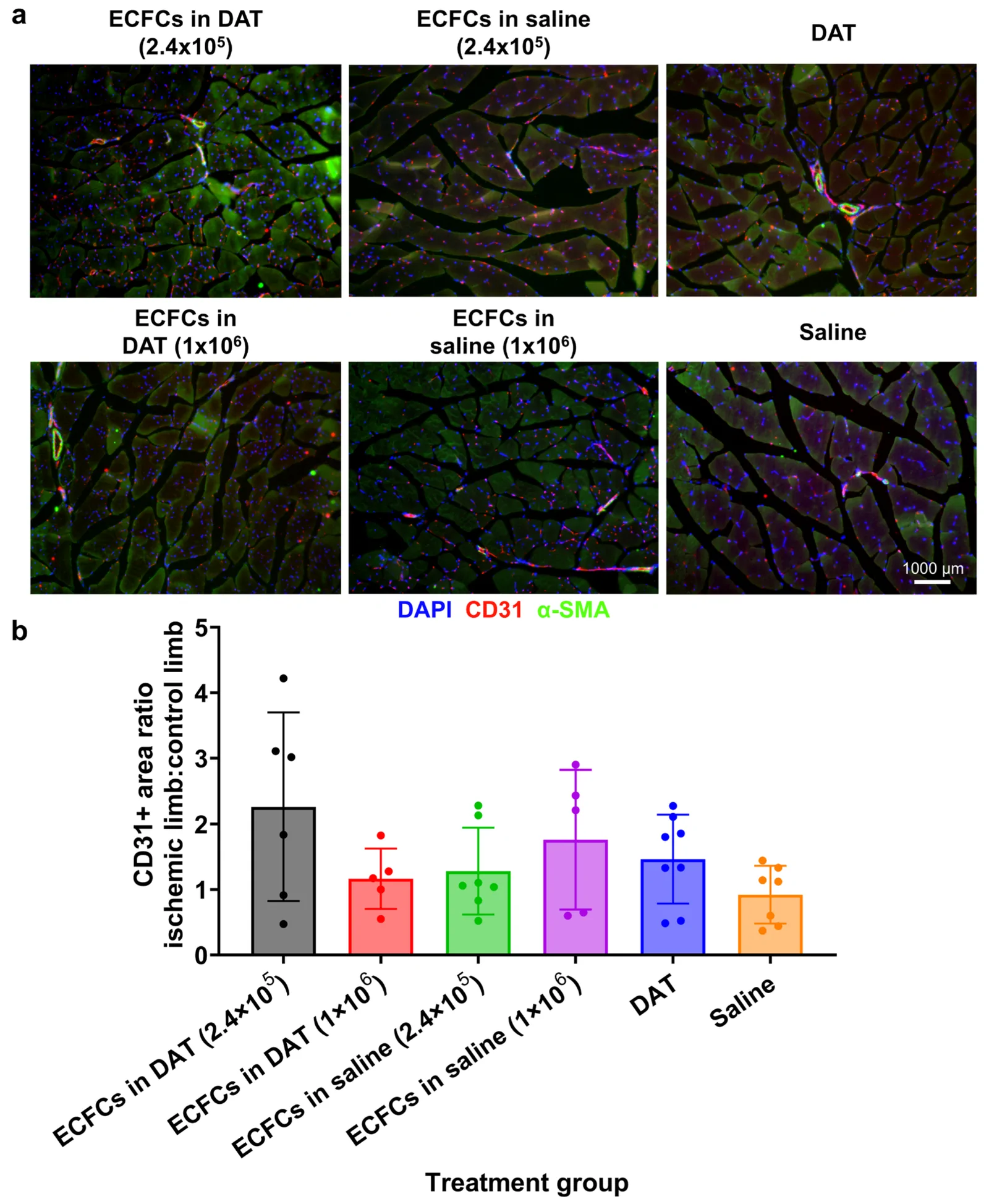
CD31^+^ capillary density in the gastrocnemius muscle on day 35 post-ligation. (**a**) Representative images of the surgical hind limb gastrocnemius muscle sections stained for CD31 (red), α-SMA (green), and DAPI for nuclei (blue). (**b**) CD31 positive pixel area/tissue section area in the surgical hindlimb, normalized to the non-surgical hindlimb, from 3–5 fields of view/section and 3 sections/muscle sample (N = 5–6 ECFC lines, n = 5–8 mice/group).

LDPI was used to monitor the recovery of blood flow in the ischemic hindlimbs in response to the treatment groups. On day 1 following surgery, perfusion was notably reduced in the ischemic (right) hindlimb (Figure 5a), and perfusion recovered gradually over the course of 5 weeks. LDPI was similar between all injected groups (Figure 5b). On day 35, CD31^+^ vascular density in hindlimb muscle tissues was quantified in situ using immunofluorescence microscopy (Figure 6a). Collectively, there were no differences in the CD31^+^ area between the treatment groups (Figure 6b). Importantly, no dT^+^ ECFCs were observed within the gastrocnemius muscle at this timepoint.

**Figure 7:**
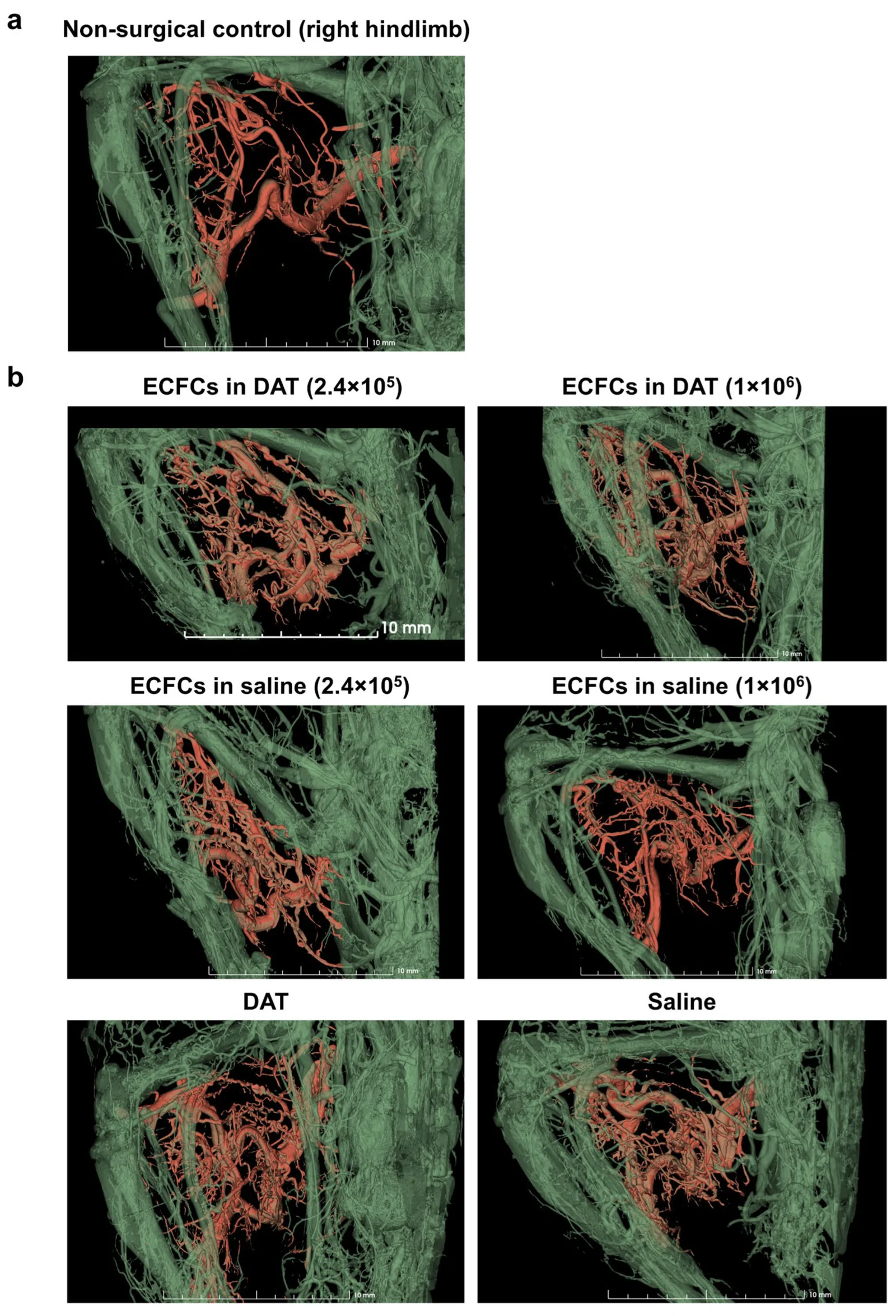
μCT angiography of mouse hindlimb vasculature at 5 weeks. Slicer image computing was used to assemble μCT scans into 3-dimensional models colored to identify perfused blood vessels located in the region of FAL surgery (orange) and perfused vessels and bones outside of the thigh region (green) in the ischemic right hindlimb. (**a**) Microscopic view of right hindlimb vasculature in a non-surgical control NOD/SCID mouse. (**b**) Microscopic view of right hindlimb vasculature in NOD/SCID mice following administration of ECFC at a low or high dose in either DAT or saline, as well as cell-free DAT and saline controls.

Finally, a pilot study using μCT angiography was performed to assess structural changes in the perfused vasculature in the hindlimbs at 35 days post-ligation (Figure S2A). Compared to blood vessels imaged in a non-surgical control mouse (Figure 7a), robust collateral remodeling characterized by the development of small tortuous blood vessels was observed around the ligation site in all treatment groups (Figure 7b). This remodeling was evident as early as 2 days following hindlimb surgery (Figure S2B), highlighting a strong arteriogenic response in NOD/SCID mice.

## 4. Discussion

Previous studies have demonstrated that ECFCs are highly proliferative in culture and can integrate into blood vessels after transplantation in vivo [30,31], making ECFCs a promising candidate for the development of cell therapies for blood vessel regeneration. However, a major challenge for any cell-based therapy is limited survival and engraftment of the transplanted cells, especially within an ischemic microenvironment [6,11]. Given the critical role of the ECM in supporting and directing cellular function, ECM-derived bioscaffolds have garnered much interest in regenerative medicine applications [12]. We previously demonstrated that human DAT-derived constructs can act as a cell- delivery platform that supports cell survival and pro-regenerative function [19,20,21]. Building on this promising foundation, we explored the delivery of human ECFCs within DAT hydrogels for the first time. Initial studies compared ECFCs cultured in the DAT hydrogels to donor-matched ECFCs cultured on TCP. Subsequently, the potential of the DAT hydrogels as an in vivo delivery platform for human ECFCs was characterized in an ischemic hindlimb model using immunodeficient NOD/SCID mice with femoral artery ligation.

Endothelial cells reside in situ within a complex microenvironment where they interact in three-dimensions with ECM as well as vascular and blood-derived cells [32]. There is growing recognition that 3D culture systems, including hydrogels, can provide a more physiologically relevant microenvironment than 2D monolayer culture on TCP [32]. Monolayer cell culture platforms are designed to promote cell expansion, where all cells have similar access to nutrients and growth factors in the media, and dead cells will detach and be removed during media changes [32]. The DAT hydrogel platform tested herein supported high initial ECFC viability following encapsulation, which was significantly diminished over the 6-day culture period. This decline may be associated with limitations in nutrient and waste exchange within the hydrogels, particularly as they became more contracted. Alternatively, this could be due to the retention of dead cells within the hydrogels which would have been washed off in the TCP group. Similarly, previous studies assessing various cell lines showed that viability in 3D was similar to that of 2D cultures during the first 1–5 days but was reduced in 3D as culture time was prolonged [33].

In addition, ECFC proliferation rates were considerably reduced over time in the DAT hydrogels compared to culture on TCP. Previous work has identified surface stiffness as an important factor affecting proliferation rates. Specifically, both vascular smooth muscle cells and MSCs displayed enhanced proliferation when cultured on stiffer collagen fibrils or polyacrylamide hydrogel coatings relative to those seeded on more compliant coatings [34,35]. While elastic moduli of DAT hydrogels were not assessed herein, Liguori et al. found the Young’s modulus of similarly processed decellularized cardiac tissue hydrogels fabricated with an ECM concentration of 20 mg/mL to be ~3 kPa [36]. This is in stark contrast to TCP, which is reported to be in the range of 1 GPa [37]. Therefore, it is possible that the higher fraction of proliferating ECFCs observed on TCP relative to the DAT hydrogels may be substrate stiffness-induced. Importantly, the observed hydrogel contraction over time will also affect the substrate stiffness. Even though the overall hydrogel contraction did not change between days 1 and 6 in vitro, there may have been differences in the ECM organization at the cellular level between the timepoints and/or the extended exposure to the contracted matrix may have had a greater effect on the cells. This may help to explain some of the phenotypic differences observed in vitro within the hydrogels between days 1 and 6.

Looking holistically at the flow cytometry analysis of the ECFC phenotypic markers, the data supports that encapsulation and culture within the 3D DAT hydrogels minimally altered the expression of cell surface markers compared to ECFCs cultured on TCP. Importantly, CD31 expression levels were high in ECFCs cultured in DAT hydrogels and on TCP, supporting that the ECFCs maintained an endothelial cell phenotype. Similarly, VEGFR2 expression, though significantly decreased on day 1 for ECFCs cultured in DAT hydrogels, was restored to similar levels to ECFCs cultured on TCP by day 6. While it is unclear what caused the transient decline in VEGFR2 expression for ECFCs cultured in DAT hydrogels on day 1, the level of expression still falls within a range that has been reported in the literature, with Joo et al. demonstrating ~75% positivity for ECFCs [38] and Guduric-Fuchs et al. demonstrating ~60% positivity for ECFCs [39]. Similarly, CD34 and CD133 expression were maintained at similar levels for ECFCs cultured in DAT hydrogels compared to those cultured on TCP. CD34 is commonly used for detection and isolation of stem and progenitor cells from hematopoietic and endothelial lineages [40], suggesting that the DAT hydrogel may be an effective platform for maintaining the progenitor phenotype in cultured ECFCs. Furthermore, previous work has reported that ECFCs with high CD34 expression exhibited higher tube-forming capacity and tip-cell gene expression compared to those with low CD34 expression [41]. In contrast, ALDH activity for ECFCs cultured in DAT hydrogels was found to be significantly decreased. ALDH is an enzyme responsible for the detoxification of aldehyde molecules, helping to protect long-lived cells from oxidative stress [42]. High ALDH activity is conserved in multiple mesodermal progenitor cell lineages [27], and ALDH activity is typically reduced as cells differentiate. Decreases in ALDH activity in ECFCs have been noted over time with in vitro expansion [43]. However, the accelerated loss of ALDH activity following culture in DAT hydrogels is interesting in light of the findings that other cell surface marker expression was largely maintained. Future studies should explore whether this change is associated with any alterations in the pro-angiogenic function of the ECFCs, including using in vitro culture assays to characterize pro-angiogenic gene expression and/or measure the capacity of the cells to stimulate tubule formation.

DAT hydrogels seeded with ECFC were subsequently tested as a delivery platform for human ECFCs, characterizing the effects of two cell doses in DAT versus saline using an FAL-induced murine model of hindlimb ischemia in NOD/SCID mice. ECFCs were transduced with luciferase to allow for multimodal cell tracking following IM delivery. Interestingly, the delivery of ECFCs in DAT hydrogels at a low dose demonstrated enhanced signal retention compared to an equal dose of ECFCs delivered in saline, with the luminescent signal appearing to plateau 1 week earlier than other groups. One possible explanation for why viable ECFC retention was augmented in the low-dose DAT hydrogel group is that, at the higher dose, the DAT may have been unable to support the cell numbers delivered into the oxygen/nutrient-deprived environment of the ischemic limb. Another possible explanation is that the higher dose of ECFCs may have triggered a more potent innate immune response that resulted in rapid cell clearance, given that NOD/SCID mice retain innate immune-cell activity including macrophages and NK cells [44]. In previous work, ASCs delivered in immunodeficient athymic *nu/nu* mice were very rapidly cleared following IM injection, with most of the retained signal being localized to macrophages via immunofluorescent co-localization, indicating the ASCs could have been targeted by macrophages [45]. In the current study, the immune response could have been exacerbated by the engineered expression of large exogenous proteins such as dT and FLuc2 [46]. NOD/SCID mice lack mature T- and B- cells and have reduced natural killer cell and macrophage function, but it may be beneficial to employ a more severely compromised mouse model such as NOD/SCID/ILRg^null^ (NSG) mice or to test the effects of macrophage depletion using clodronate to further understand the mechanisms of ECFC clearance.

Notably, enhanced ECFC retention in the low-dose DAT group did not translate to enhanced recovery of limb perfusion or changes in CD31^+^ vessel density after 35 days in vivo. No dTomato^+^ ECFCs were detected within any of the tissue sections visualized in the immunofluorescence staining, suggesting that the transplanted cells did not effectively engraft into the murine capillaries. It is possible that a small subset of ECFCs incorporated into the host vasculature but were simply not observed histologically. However, when combined with the BLI data, these findings suggest that ECFCs do not persist for long periods; therefore, they are not major contributors to the stable vasculature in this model. As direct integration into the vascular system is postulated to be the primary mechanism of action for ECFCs, limited engraftment is consistent with the lack of functional improvement observed in the FAL-CLI model.

Previous studies have reported that ECFCs alone are unable to mediate functional recovery of hindlimb ischemia within mice when administered without supportive accessory cells. For example, Kang et al showed that the intraperitoneal delivery of up to 2 × 10^6^ Matrigel-encapsulated human ECFCs without supportive MSCs did not improve blood flow recovery in ischemic hind limbs until day 28 of the study [47]. Another study found that the intramuscular delivery of 1 × 10^5^ human ECFCs in saline did not improve recovery of limb perfusion over 28 days [48]. Other studies that have demonstrated the ability of ECFCs to improve perfusion have differed in their cell delivery strategies. For example, Goto et al. (2016) showed that ECFCs could independently improve limb reperfusion when ECFCs were transduced to overexpress integrin β1 and systemically injected into the circulation, but not when they were locally injected into the ischemic muscle [48].

Most importantly, there is considerable evidence to support that a combined cell therapy approach may be more effective. The study by Kang et al. found that when the ECFCs were delivered in combination with human mesenchymal progenitor cells, blood flow was significantly recovered as early as day 5 following injection and was maintained until day 28 [47]. Similarly, when human ECFCs were co-delivered intramuscularly with human MSCs in a 3D spheroid in a *nu/nu* mice with FAL-CLI, there was significantly improved blood flow in the ischemic limb compared to ECFCs delivered alone [49]. Because ECFCs co-delivered subcutaneously with MSCs demonstrated greater survival and proliferation than ECFCs delivered alone, the authors concluded that the MSCs augmented survival of the ECFCs and increased the efficiency of engraftment.

Given the growing evidence that multiple cell types may be required for robust and lasting blood vessel network formation, future work should be directed towards the incorporation of additional cell types into the DAT hydrogels in a stepwise and cell dose-dependent fashion for co-delivery into preclinical models of CLI. In particular, MSCs that possess a pro-angiogenic secretome and can act as vessel-supportive perivascular cells may be useful to stabilize newly formed vessels [50]. Recent work in our lab assessing the combined delivery of human ASCs and ECFCs in 3D implantable DAT scaffolds found that co-delivery enhanced viable ECFC retention following subcutaneous implantation, supporting the potential benefit of combination cell delivery within DAT-based bioscaffolds [51].

Finally, a pilot study to visualize vessel regeneration following cell delivery demonstrated a robust arteriogenic response in the NOD/SCID FAL model irrespective of treatment group. This response was characterized by collateral remodeling of blood vessels around the surgical site, allowing the distal portion of the femoral artery and vein to be partially re-perfused, fed by the formation of many tortuous vessels that were not visible in the non-surgical limb. Importantly, this robust recovery in all groups may further explain the absence of a response to implanted ECFCs. In a system where collateral vessel formation is not limited by endothelial abundance or growth potential, the addition of endothelial precursors may not have a substantial effect on recovery.

While the FAL-induced murine model of hindlimb ischemia is the predominant preclinical mouse model for testing cell therapies targeting peripheral artery disease and CLI [52], there are some key differences from the clinical presentation of CLI in humans. The FAL-CLI model features an acute and non-sustained ischemia, with mice lacking clinically important comorbidities given their young age and good health upon surgery and cell delivery [53]. Given the strong baseline recovery of perfusion in the saline-treated control mice, the use of a more clinically representative hindlimb ischemia model may reveal further beneficial effects of ECFCs for cell therapy. Recently, a two-stage model of murine hindlimb ischemia has been developed, involving femoral artery occlusion followed by full excision of the femoral artery at day 14. This model demonstrated longer and more sustained functional impairment than traditional and more acute FAL-models [54]. Additionally, some recent studies have employed FAL in mice with streptozotocin-induced hyperglycemia to model impaired arteriogenic processes seen in patients with diabetes mellitus [55]. These adaptations to the FAL-CLI model should be considered as more clinically relevant directions for future work. In particular, the use of DAT hydrogels for the co-delivery of ECFCs and other supportive cell types within these models may help enable better clinical translation of combinatorial cell delivery.

## 5. Conclusions

In summary, this work investigated the use of a clinically translatable biomaterial as a delivery vehicle for ECFCs with the goal of promoting cellular retention and improved outcomes in a murine hindlimb ischemia model. Initial in vitro data suggested that the DAT hydrogels supported ECFCs in culture, with reductions in cell viability and proliferation and without a major effect on cell phenotype relative to cells cultured on TCP. When delivered at a lower density, delivery in the DAT hydrogels enhanced ECFC retention compared to delivery in saline. However, this increased persistence was not associated with improved outcomes in vivo. Moreover, ECFCs were not observed to have integrated into the vasculature in any of the groups explored, which may have contributed to the lack of functional improvements in the FAL-CLI model. Future work should investigate incorporating additional strategies, such as co-delivery and/or preconditioning to enhance ECFC integration into the host tissues and assess whether this can augment functional vascular regeneration. Further, the µCT data highlights the robust healing response in the FAL-CLI model employed through collateral vessel formation. To better mimic the human condition, future studies are required to develop more clinically relevant models with impaired vascular recovery, potentially making use of aged or diabetic mice to better reflect the patient population impacted by PAD/CLI.

## Data Availability

The raw data supporting the conclusions of this manuscript will be made available by the authors, without undue reservation, to any qualified researcher.

## Supplementary Materials

The following supporting information can be downloaded at: https://www.mdpi.com/article/10.3390/jfb17080368/s1, Figure S1: Lentiviral transduction did not alter ECFC viability, proliferation, or CD31 expression; Figure S2: NOD/SCID mice demonstrate robust and rapid vascular regeneration following FAL surgery.

## Abbreviations

The following abbreviations are used in this manuscript:

CLI	Critical limb ischemia
ECFCs	Endothelial colony-forming cells
DAT	Decellularized adipose tissue
PAD	Peripheral arterial disease
T2D	Type 2 diabetes
ECM	Extracellular matrix
ASCs	Adipose-derived stromal cells
ALDH	Aldehyde dehydrogenase
NOD/SCID	Non-obese diabetic/severe combined immune deficiency
LDPI	Laser doppler perfusion imaging
BLI	Bioluminescent imaging
TCP	Tissue culture polystyrene
