# Supplementary material for: Human Decellularized Adipose Tissue Hydrogels as a Delivery Platform to Enhance Human Endothelial Colony-Forming Cell Retention and Vascular Regeneration
Source: J Funct Biomater. 2026 Jul 30;17(8):368. doi: 10.3390/jfb17080368 (PMC13513861; doi:10.3390/jfb17080368)
Supplement: Supplementary file 1 [file jfb-17-00368-s001.zip › jfb-4369204-supplementary.pdf]

Supplemental information

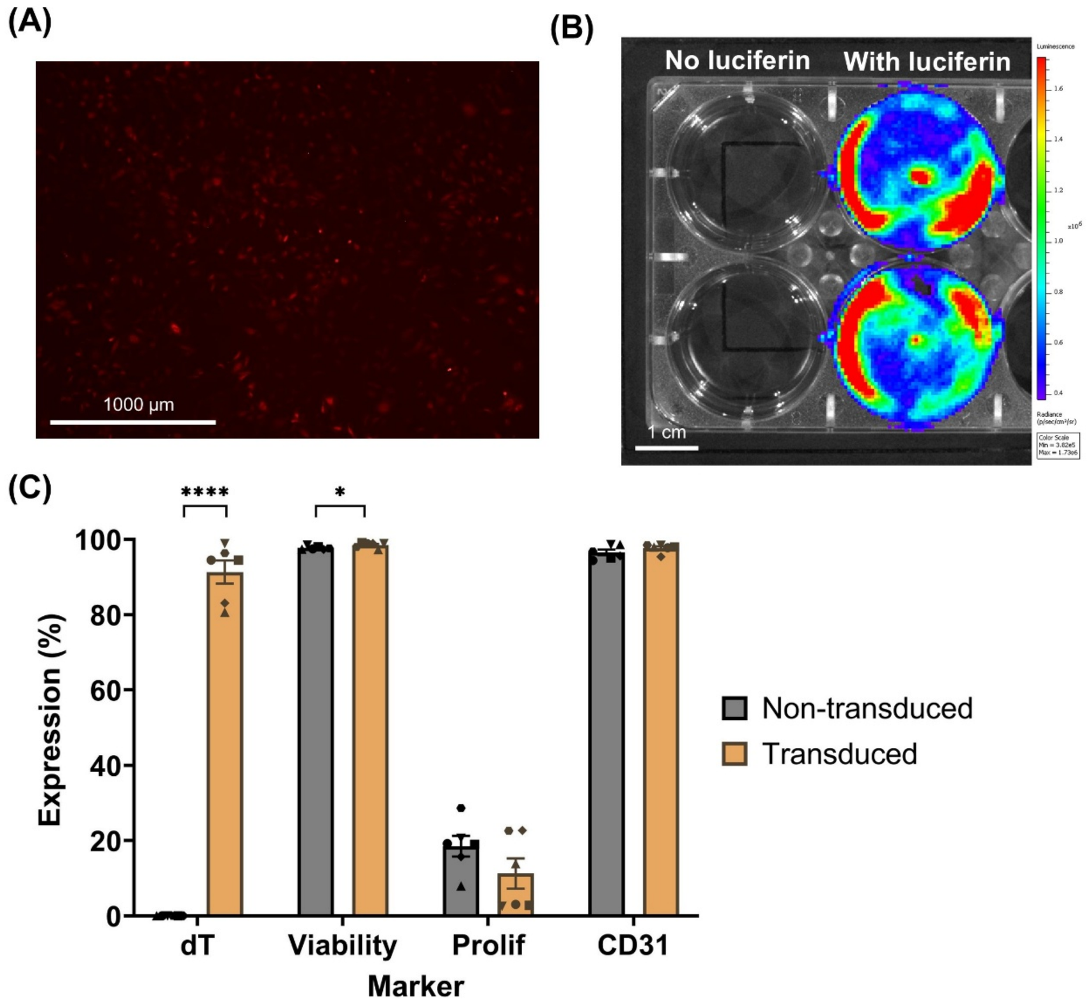

Figure S1. Lentiviral transduction did not alter ECFC viability, proliferation, or CD31 expression. Human ECFCs were transduced using lentiviral vectors to stably co-express **(A):** dT and **(B):** firefly luciferase. **(C):** dT expression, ECFC viability (Zombie Yellow), ECFC proliferation (EdU incorporation), and CD31 expression were unchanged following transduction. *$p<0.05$, ****$p<0.0001$.

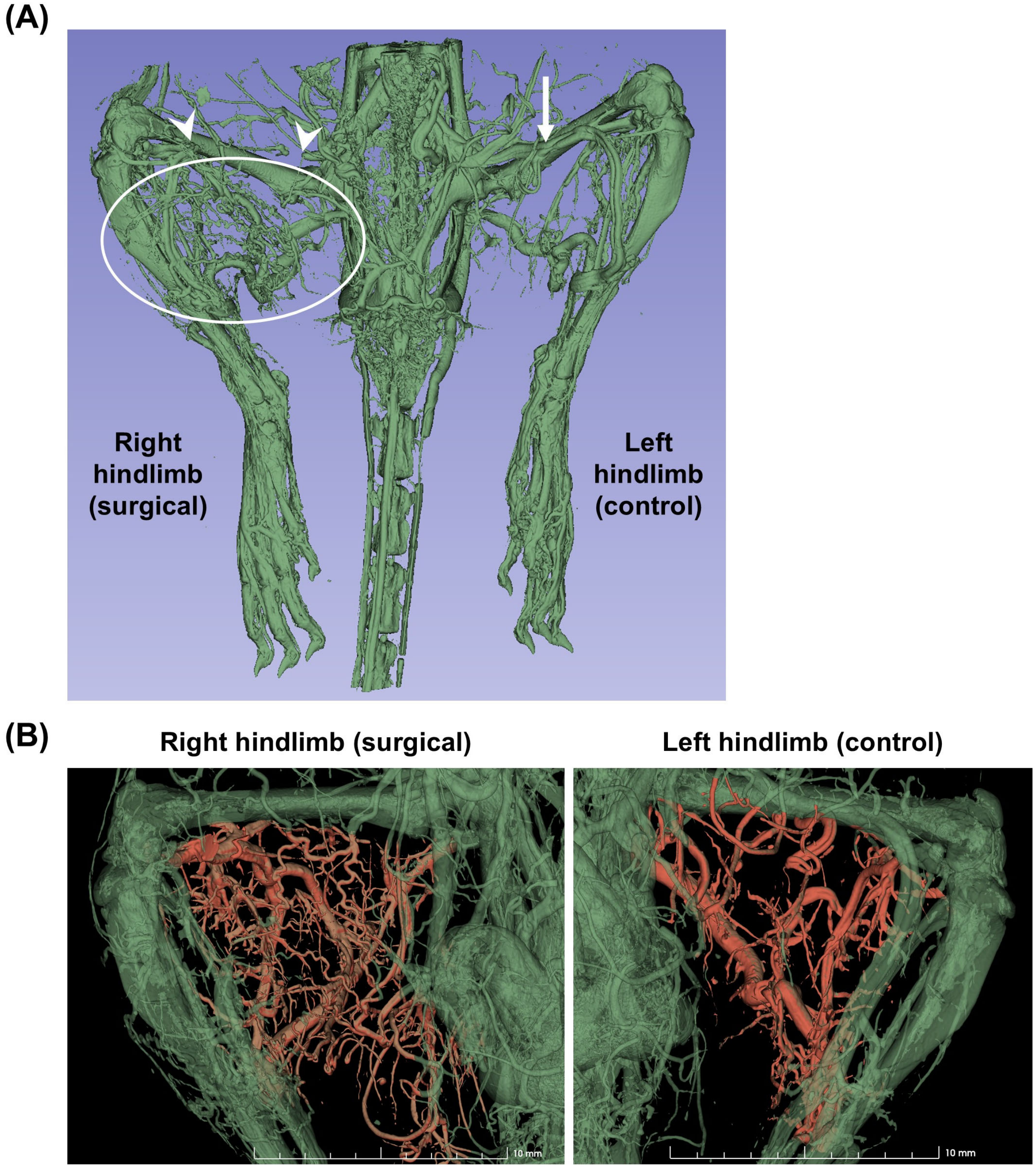

Figure S2. NOD/SCID mice demonstrate robust and rapid vascular regeneration following FAL surgery. (A): Macroscopic μCT angiography of mouse hindlimb vasculature 35 days after FAL surgery. White arrow depicts the paired femoral artery and vein in the left hindlimb (non-surgical control). White arrowheads depict the two sites where the femoral artery and vein were ligated, and the central portion was excised during surgery. The white circle denotes large, unorganized blood vessel mass that forms following FAL surgery in the right hindlimb. **(B):** Right (surgical) and left (non-surgical) hindlimb in a NOD/SCID mouse 2 days following FAL surgery.
